# Human cytomegalovirus mediates APOBEC3B relocalization early during infection through a ribonucleotide reductase-independent mechanism

**DOI:** 10.1128/jvi.00781-23

**Published:** 2023-08-11

**Authors:** Elisa Fanunza, Adam Z. Cheng, Ashley A. Auerbach, Bojana Stefanovska, Sofia N. Moraes, James R. Lokensgard, Matteo Biolatti, Valentina Dell'Oste, Craig J. Bierle, Wade A. Bresnahan, Reuben S. Harris

**Affiliations:** 1 Department of Biochemistry and Structural Biology, University of Texas Health San Antonio, San Antonio, Texas, USA; 2 Department of Life and Environmental Sciences, University of Cagliari, Cittadella Universitaria di Monserrato, Cagilari, Italy; 3 Department of Biochemistry, Molecular Biology and Biophysics, University of Minnesota, Minneapolis, Minnesota, USA; 4 Howard Hughes Medical Institute, University of Texas Health San Antonio, San Antonio, Texas, USA; 5 Department of Medicine, University of Minnesota, Minneapolis, Minnesota, USA; 6 Department of Public Health and Pediatric Sciences, University of Turin, Turin, Italy; 7 Department of Pediatrics, Division of Pediatric Infectious Diseases and Immunology, University of Minnesota, Minneapolis, Minnesota, USA; 8 Department of Microbiology and Immunology, University of Minnesota, Minneapolis, Minnesota, USA; The University of Arizona, Tucson, Arizona, USA

**Keywords:** APOBEC3B (A3B), herpesviruses, human cytomegalovirus, immediate-early genes, innate immunity, ribonucleotide reductase

## Abstract

The APOBEC3 family of DNA cytosine deaminases comprises an important arm of the innate antiviral defense system. The gamma-herpesviruses Epstein-Barr virus and Kaposi’s sarcoma-associated herpesvirus and the alpha-herpesviruses herpes simplex virus (HSV)-1 and HSV-2 have evolved an efficient mechanism to avoid APOBEC3 restriction by directly binding to APOBEC3B and facilitating its exclusion from the nuclear compartment. The only viral protein required for APOBEC3B relocalization is the large subunit of the ribonucleotide reductase (RNR). Here, we ask whether this APOBEC3B relocalization mechanism is conserved with the beta-herpesvirus human cytomegalovirus (HCMV). Although HCMV infection causes APOBEC3B relocalization from the nucleus to the cytoplasm in multiple cell types, the viral RNR (UL45) is not required. APOBEC3B relocalization occurs rapidly following infection suggesting the involvement of an immediate early or early (IE/E) viral protein. In support of this possibility, genetic (IE1 mutant) and pharmacologic (cycloheximide) strategies that prevent the expression of IE/E viral proteins also block APOBEC3B relocalization. In comparison, the treatment of infected cells with phosphonoacetic acid, which interferes with viral late protein expression, still permits A3B relocalization. These results combine to indicate that the beta-herpesvirus HCMV uses an RNR-independent, yet phenotypically similar, molecular mechanism to antagonize APOBEC3B.

**IMPORTANCE:**

Human cytomegalovirus (HCMV) infections can range from asymptomatic to severe, particularly in neonates and immunocompromised patients. HCMV has evolved strategies to overcome host-encoded antiviral defenses to achieve lytic viral DNA replication and dissemination and, under some conditions, latency and long-term persistence. Here, we show that HCMV infection causes the antiviral factor, APOBEC3B, to relocalize from the nuclear compartment to the cytoplasm. This overall strategy resembles that used by related herpesviruses. However, the HCMV relocalization mechanism utilizes a different viral factor(s) and available evidence suggests the involvement of at least one protein expressed at the early stages of infection. This knowledge is important because a greater understanding of this mechanism could lead to novel antiviral strategies that enable APOBEC3B to naturally restrict HCMV infection.

## INTRODUCTION

The APOBEC3 (A3) system is an essential part of the cellular innate immune response to viral infections [reviewed by Green and Weitzman (1), Harris and Dudley (2), and Hakata and Miyazawa (3)]. A3-mediated restriction has been reported for a broad number of DNA-based viruses, including exogenous viruses (retroviruses, polyomaviruses, papillomaviruses, parvoviruses, hepadnaviruses, and herpesviruses) and endogenous viruses and transposable elements. The mechanism by which virus restriction occurs is well documented and dependent partly on the ability of A3 enzymes to introduce mutations in the viral genome by catalyzing cytosine deamination in exposed single-stranded (ss)DNA intermediates. In addition, deaminase-independent antiviral activity has been reported against endogenous retroelements, reverse-transcribing viruses, adeno-associated viruses, and RNA viruses, and this may be attributed to strong nucleic acid binding activity.

The continuous arms race between host and viruses leads to the selection of viral factors that are able to counteract innate immune factors, including the A3 antiviral enzymes. For example, HIV-1, HIV-2, and related lentiviruses encode a viral accessory protein Vif that mediates the degradation of restrictive A3s (4, 5). Recently, a novel mechanism of A3 counteraction was discovered for the gamma-herpesviruses Epstein-Barr virus (EBV), which uses the viral ribonucleotide reductase (RNR) large subunit, BORF2, to directly bind, inhibit, and relocalize APOBEC3B (A3B) from the nucleus to the cytoplasm, thus preserving viral genome integrity (6). This mechanism of A3 neutralization is likely to be conserved because at least two other herpesviruses, Kaposi’s sarcoma-associated herpesvirus (KSHV) and herpes simplex virus 1 (HSV-1), whose RNRs [open reading frame (ORF) 61 and ICP6, respectively] physically interact with A3B, as well with APOBEC3A (A3A), and trigger their redistribution from the nucleus to the cytoplasmic compartment (7 - 10). In further support of evolutionary conservation, a systematic analysis of a large panel of present-day gamma-herpesvirus RNRs and primate A3B proteins indicates that the evolution of this viral RNR-mediated A3B neutralization mechanism was likely selected by the birth of the *A3B* gene by unequal crossing-over in an ancestral Old World primate approximately 29–43 million years ago (8, 11).

Human cytomegalovirus (HCMV) is a member of the beta-herpesvirus subfamily. HCMV is a ubiquitous virus, found in approximately 90% of the worldwide population. HCMV infection is usually asymptomatic in healthy individuals, but it can cause severe disease in immunocompromised hosts [reviewed by Tyl et al. (12) and Griffiths and Reeves (13)]. Congenital HCMV infections are also a leading cause of birth defects [reviewed by Manicklal et al. (14) and Britt (15)]. HCMV has a large double-stranded (ds)DNA genome of 235  kb—the largest among known human herpesviruses—containing 165 canonical ORFs and several alternative transcripts [reviewed by Turner and Mathias (16)]. Lytic HCMV infection involves a temporal cascade of gene expression. A small subset of genes, termed immediate-early (IE) genes, are the first to be expressed. Transcription of IE genes does not require *de novo* protein synthesis. IE proteins together with host factors mediate the expression of the kinetically distinct early genes (E), whose products in large part promote viral genome replication and the expression of late genes (L) [reviewed by Turner and Mathias (16)].

Several HCMV gene products have acquired the ability to subvert different signaling pathways and modulate various components of the immune response to make the host cell more permissive to viral replication and survival [reviewed by Biolatti et al. (17) and Patro (18)]. Given the ability of gamma- and alpha-herpesviruses (EBV/KSHV and HSV-1/2, respectively) to inhibit A3B, we sought to investigate whether HCMV possesses a similar RNR-mediated A3 neutralization mechanism. Our results demonstrate that HCMV infection is also capable of inducing the selective nuclear to cytoplasmic relocalization of A3B. However, surprisingly, results with multiple independent viral strains and cell lines indicate that the relocalization mechanism of A3B by HCMV is not conserved with other human herpesviruses and, instead, occurs independently of the HCMV UL45 RNR. In addition to this strong mechanistic distinction, multiple lines of evidence including rapid A3B relocalization kinetics suggest involvement of at least one viral IE-E protein in A3B relocalization.

## RESULTS

### HCMV mediates A3B relocalization independently of viral strain and cell type

**Fig 1:**
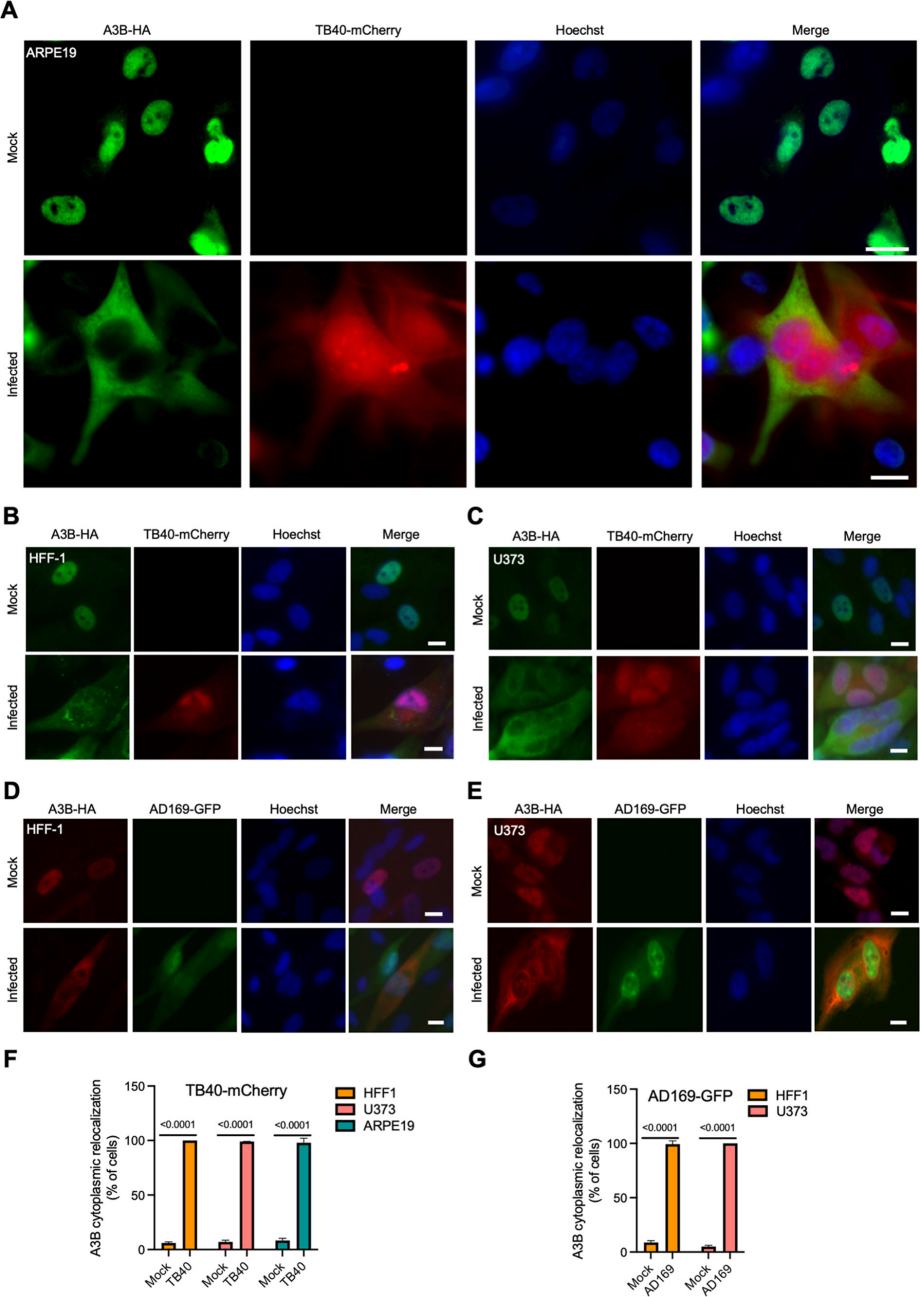
A3B relocalization occurs with multiple HCMV strains in different cell types. (A–E) Representative IF microscopy images of the indicated cell types stably expressing A3B-HA incubated with medium alone (mock) or infected with the indicated HCMV strains for 72 h (10 µm scale). High-magnification images in Fig. 1A were taken with a Nikon Eclipse Ti2 system, and all other images were taken with an EVOS cell imaging system. (**F and G**) Quantification of A3B-HA subcellular localization phenotypes shown in panels A–E. Each histogram bar reports the percentage of cells with cytoplasmic A3B-HA (*n* > 100 cells per condition; mean ± SD with indicated *P* values from unpaired Student’s *t*-tests).

We previously reported the ability of gamma- and alpha-herpesviruses to bind to A3B and mediate its relocalization from the nuclear compartment into cytoplasmic aggregates (6 - 8). To investigate whether the beta-herpesvirus HCMV has similar functionality, immunofluorescence (IF) microscopy experiments were done using infected human retinal pigment epithelial cells, ARPE19. First, ARPE19 cells were stably transduced with a lentivirus expressing C-terminally HA-tagged A3B. As reported for other human cell types (11, 19 - 21), A3B localizes primarily to the nuclear compartment of mock/non-infected ARPE19 cells (representative image in Fig. 1A). Next, ARPE19 transduced cells were infected with HCMV strain TB40/E that expresses the mCherry protein (TB40-mCherry) and analyzed for A3B localization by IF microscopy 72 hours post-infection (hpi). Infected, mCherry-positive cells are visibly enlarged, as expected for productive cytomegalovirus infection, and A3B becomes predominantly cytoplasmic (representative image in Fig. 1A and quantification in Fig. 1F). TB40-mCherry infection also causes A3B-HA relocalization in other cell types including primary human foreskin fibroblast cells (HFF-1) and the human glioma cell line U373 (Fig. 1B and C and quantification in Fig. 1F). Moreover, when ARPE19 cells were transfected to express each of the seven different human A3 family members, A3B is the only protein to show a major change in subcellular distribution following HCMV infection (Fig. S1).

To ask whether the A3B relocalization mechanism extends to other HCMV strains, HFF-1 and U373 stably transduced with HA-tagged A3B were infected with the laboratory-adapted GFP-expressing AD169 strain (AD169-GFP), and IF microscopy was done 72 hpi. As mentioned above, AD169-GFP infection induces strong relocalization of A3B from the nuclear compartment to the cytoplasm (Fig. 1D and E and quantification in Fig. 1G). Similar A3B-HA relocalization is observed during infection of HFF-1 cells with the Merlin strain (Fig. S2A). As an additional control for specificity, A3B-EGFP but not EGFP alone relocalizes to the cytoplasmic compartment following infection of ARPE19 cells with TB40-mCherry (Fig. S2B). Thus, the A3B relocalization phenotype is evident following infection with multiple HCMV strains and in a range of different cell types (both primary and immortalized) permissive for HCMV infection.

### Catalytic mutant and endogenous A3B are relocalized upon HCMV infection

**Fig 2:**
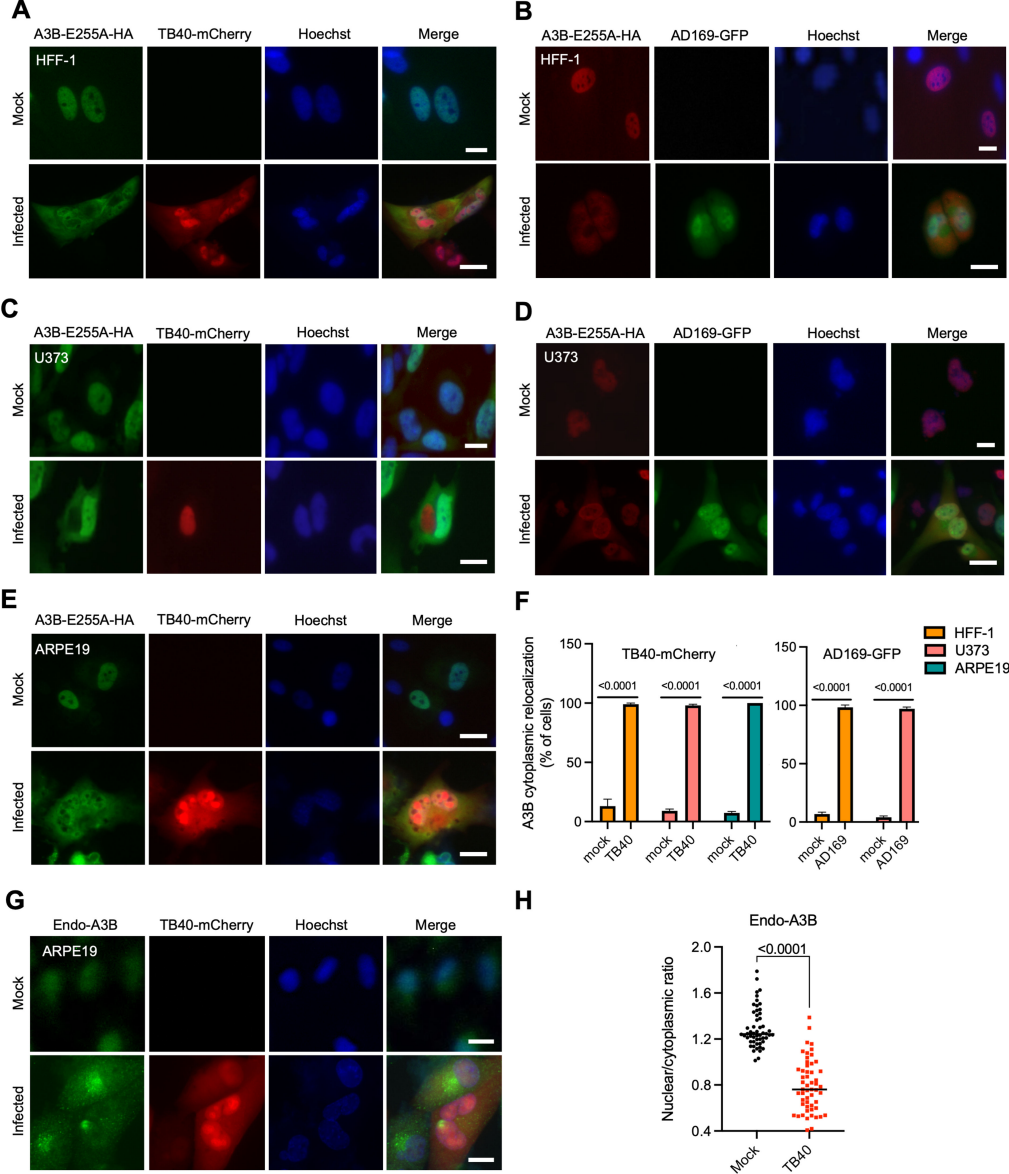
Catalytic mutant and endogenous A3B are relocalized by HCMV. (A–E) Representative IF microscopy images of the indicated cell types stably expressing A3B-E255A-HA incubated with medium alone (mock) or infected with the indicated HCMV strains for 72 h (10 µm scale). (**F**) Quantification of A3B-E255A-HA subcellular localization phenotypes shown in panels A–E. Each histogram bar reports the percentage of cells with cytoplasmic A3B-HA (*n* > 100 cells per condition; mean ± SD with indicated *P* values from unpaired Student’s *t*-tests). (**G**) Representative IF microscopy images of ARPE19 cells incubated with medium alone (mock) or infected with TB40-mCherry for 72 h and then stained for endogenous A3B (10 µm scale). (**H**) Quantification of endogenous A3B subcellular localization phenotype shown in panel G. The dot-plot chart shows the ratio between nuclear and cytoplasmic fluorescence intensity (*n* > 50 cells per condition; *P* values obtained with unpaired Student’s *t*-tests).

Overexpression of wildtype (wt) A3B causes chromosomal DNA deamination, strong DNA damage responses, cell cycle perturbations, and eventually cell death (22 - 24). These phenotypes require the catalytic activity of A3B. To address the possibility that A3B relocalization may be triggered indirectly by one of these events, HFF-1, U373, and ARPE19 cells were transduced with a lentiviral construct expressing the catalytically inactive A3B mutant (A3B-E255A). HCMV infection and IF microscopy experiments were done as above. In all instances, A3B-E255A relocalizes from the nucleus to the cytoplasm following infection with TB40-mCherry or AD169-GFP (representative images in Fig. 2A through E and quantification in Fig. 2F). Importantly, the magnitude of relocalization was indistinguishable for wt A3B and A3B-E255A (Fig. S3A). These results demonstrate that the relocalization of A3B occurs independent of its DNA deamination activity and is unlikely to be part of a general DNA damage response.

To further confirm that the relocalization phenotype is not a general effect of A3B overexpression, we next evaluated the subcellular localization of the endogenous protein. ARPE19 cells were infected with TB40-mCherry, allowing 72 h for infection to progress, and then performing IF microscopy with the rabbit anti-human A3B monoclonal antibody 5210-87-13 (25). As observed above with overexpressed A3B-HA (with or without catalytic activity), the endogenous A3B protein also shows strong relocalization from the nucleus to the cytoplasm (Fig. 2G and H). These results indicate that the A3B relocalization mechanism of HCMV is not likely to be an artifact of protein overexpression because endogenous A3B also exhibits a clear relocalization phenotype following virus infection.

### HCMV UL45 is incapable of binding, inhibiting, or relocalizing human A3B

**Fig 3:**
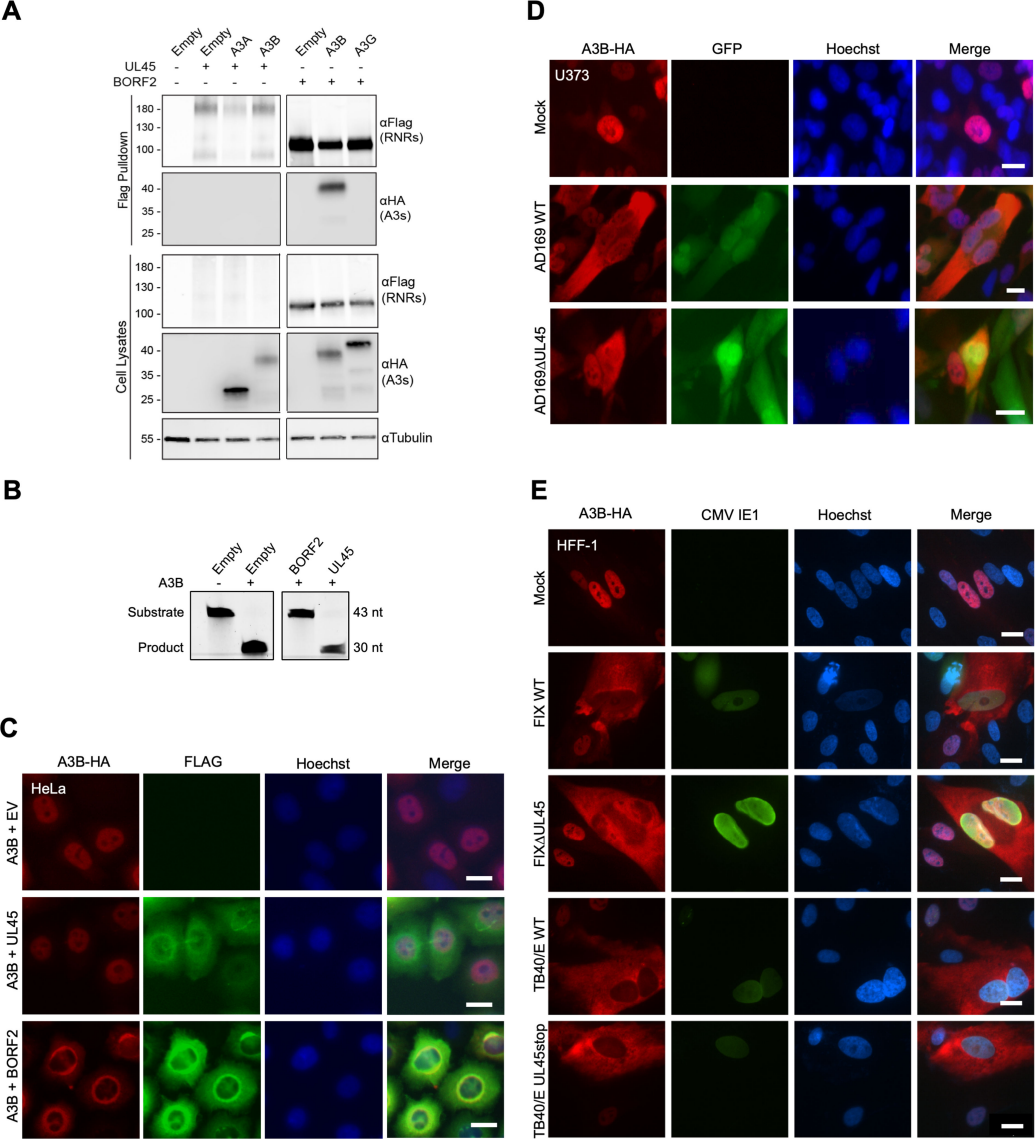
A3B relocalization is UL45 independent. (**A**) Co-IP of transfected HCMV UL45-FLAG with the indicated A3-HA constructs in 293T cells. Cells co-transfected with EBV BORF2 and A3B or A3G are used as positive and negative controls, respectively. (**B**) TBE-urea PAGE analysis of A3B deaminase activity in the presence of empty vector, HCMV UL45, or EBV BORF2. (**C**) Representative IF microscopy images of HeLa cells transiently expressing A3B-HA together with empty vector, HCMV UL45-FLAG, or EBV BORF2-FLAG (10 µm scale). (**D and E**) Representative IF microscopy images of the indicated cell types stably expressing A3B-HA incubated with medium alone (mock) or infected with the indicated HCMV strains and UL45-null derivatives for 72 h (10 µm scale).

The only gamma- and alpha-herpesvirus protein required for A3B relocalization is the large subunit of the viral RNR (6 - 8). The large RNR subunit of EBV, BORF2, directly binds A3B, inhibits its catalytic activity, and relocalizes the protein from the nucleus to the cytoplasm. To address whether the HCMV large RNR subunit, UL45, is capable of similarly binding to A3B, we performed a series of coimmunoprecipitation (co-IP) experiments. 293T cells were transfected with empty vector or FLAG-tagged HCMV UL45 or EBV BORF2 together with a HA-tagged human A3B or other A3 constructs as negative controls. As expected, EBV BORF2 robustly co-IPs A3B but not the negative control A3G (Fig. 3A). In parallel experiments, HCMV UL45 appears incapable of co-IP of either A3B or A3A (Fig. 3A). However, conclusions from these experiments are limited by relatively low UL45 expression levels in cell extracts, and multiple expressed products including likely monomeric and dimeric forms (full-length UL45 is predicted to be ~108 kDa).

We, therefore, turned to other approaches to ask whether HCMV UL45 is capable of interfering with A3B catalytic activity. First, 293T cells were transfected with human A3B, together with empty vector, HCMV UL45, or EBV BORF2. 48 h post-transfection, whole cell lysates were incubated with a fluorescently labeled ssDNA substrate containing a single 5′-TC deamination motif. If A3B catalyzes the deamination of this cytosine to uracil, cellular uracil N-DNA glycosylase 2 excises the uracil and the resulting abasic site is cleaved by sodium hydroxide, leading to the formation of a short product oligonucleotide. Consistent with previous results (6), A3B exhibits robust ssDNA C-to-U activity in cell extracts, and its activity is strongly inhibited by BORF2 (Fig. 3B). In comparison, HCMV UL45 co-expression has a negligible effect on the ssDNA C-to-U activity of A3B in cell extracts (Fig. 3B). Next, IF microscopy experiments were done by cotransfecting HeLa cells with A3B-HA and viral RNR-FLAG constructs, allowing 48 h for expression, and imaging with specific antibodies. In contrast to EBV BORF2, which relocalizes A3B from the nuclear to the cytoplasmic compartment, the expression of HCMV UL45 has no effect on A3B subcellular localization (Fig. 3C). Taken together, negative results from co-IP, deaminase inhibition, and colocalization experiments indicate that the large RNR subunit of HCMV, UL45, is incapable of affecting the subcellular localization or ssDNA deamination activity of A3B.

To directly ask whether HCMV UL45 is required for A3B relocalization, we compared the subcellular localization phenotypes of A3B in U373 cells following infection by AD169-GFP or a derivative virus engineered to lack UL45 [AD169-GFP ΔUL45 (26)]. U373 cells were stably transduced with HA-tagged A3B 48 h prior to mock infection or infection with AD169-GFP or AD169-GFP ΔUL45. After 72 h of infection, cells were fixed, permeabilized, and imaged by IF microscopy. As described above, infection by AD169-GFP causes the relocalization of A3B from the nuclear to the cytoplasmic compartment (Fig. 3D). As expected, cells infected with AD169-GFP ΔUL45 show an indistinguishable A3B cytoplasmic relocalization phenotype (Fig. 3D and quantification in Fig. S3B). This key result was confirmed by IF microscopy experiments using two other HCMV strains (TB40/E and FIX) and otherwise isogenic UL45-null derivatives (TB40/E ΔUL45 and FIX ΔUL45) (Fig. 3E). These results demonstrate that UL45 is dispensable for HCMV-mediated relocalization of A3B and, together with the results above, that this beta-herpesvirus does not share the RNR-dependent mechanism of gamma- and alpha-herpesviruses.

### The N-terminal domain of A3B is sufficient for HCMV-mediated relocalization

**Fig 4:**
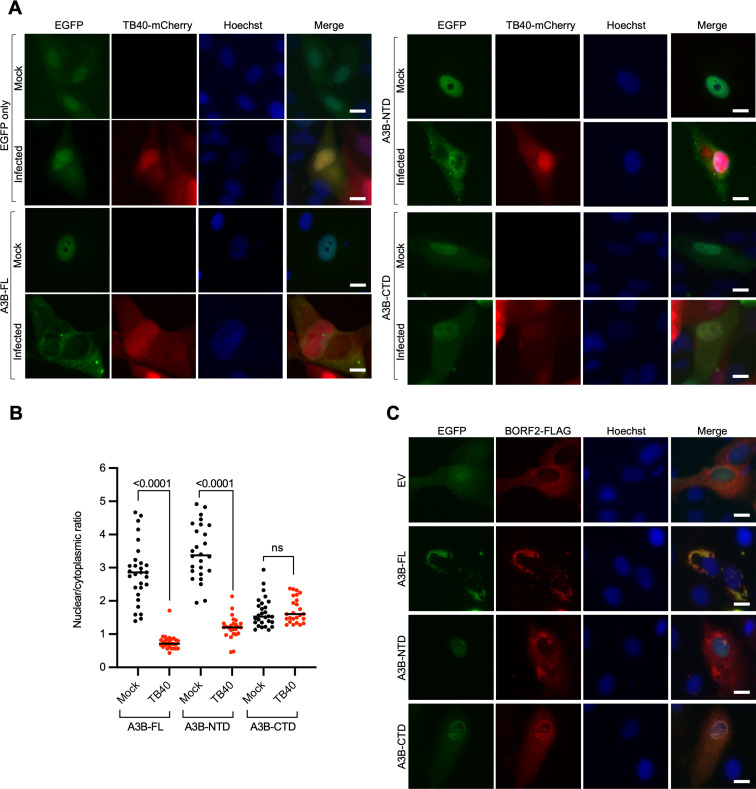
The NTD of A3B is sufficient for A3B relocalization mediated by HCMV. (**A**) Representative IF microscopy images of ARPE19 cells transiently expressing EGFP alone, A3B-FL-EGFP, A3B-NTD-EGFP, and A3B-CTD-EGFP, incubated with medium alone (mock) or infected with TB40-mCherry for 72 h (10 µm scale). (**B**) Quantification of A3B-FL, A3B-NTD, and A3B-CTD subcellular localization phenotype shown in panel A. The dot-plot chart shows the ratio between nuclear and cytoplasmic fluorescence intensity (*n* > 25 cells per condition; *P* values obtained with unpaired Student’s *t*-tests). (**C**) Representative IF microscopy images of HeLa cells transiently expressing EBV BORF2-FLAG together with EGFP alone, A3B-FL-EGFP, A3B-NTD-EGFP, and A3B-CTD-EGFP (10 µm scale).

A3B is comprised of two conserved deaminase domains: an inactive N-terminal domain (A3B-NTD) and a catalytically active C-terminal domain (A3B-CTD) (9, 27). A3B-NTD is thought to be regulatory in nature and is alone sufficient for nuclear localization (11, 19). EBV BORF2 mediates A3B relocalization by binding to the CTD and not to the NTD (6). To ask whether domain requirements might further distinguish the A3B relocalization mechanism of HCMV, IF microscopy experiments were done with ARPE19 cells transfected with EGFP-tagged full-length A3B (A3B-FL), A3B-NTD, or A3B-CTD constructs. After 72 h infection with TB40-mCherry, A3B-FL shows clear relocalization to the cytoplasmic compartment in comparison to the unchanged cell-wide EGFP control (Fig. 4A and B). Surprisingly, A3B-NTD, which shows nuclear localization in mock-infected cells, becomes predominantly cytoplasmic after infection (Fig. 4A and B). A3B-CTD has a cell-wide localization pattern that is not changed by virus infection (Fig. 4A and B). In contrast, EBV BORF2 has no effect on A3B-NTD nuclear localization, and it strongly promotes the relocalization of A3B-FL and A3B-CTD into cytoplasmic aggregates (Fig. 4C). These data combine to show that A3B-NTD is sufficient for A3B subcellular redistribution during HCMV infection and additionally distinguish the molecular mechanism from that mediated by the large RNR subunit of gamma- and alpha-herpesvirus.

### A3B relocalization occurs early during infection and requires *de novo* HCMV protein expression

**Fig 5:**
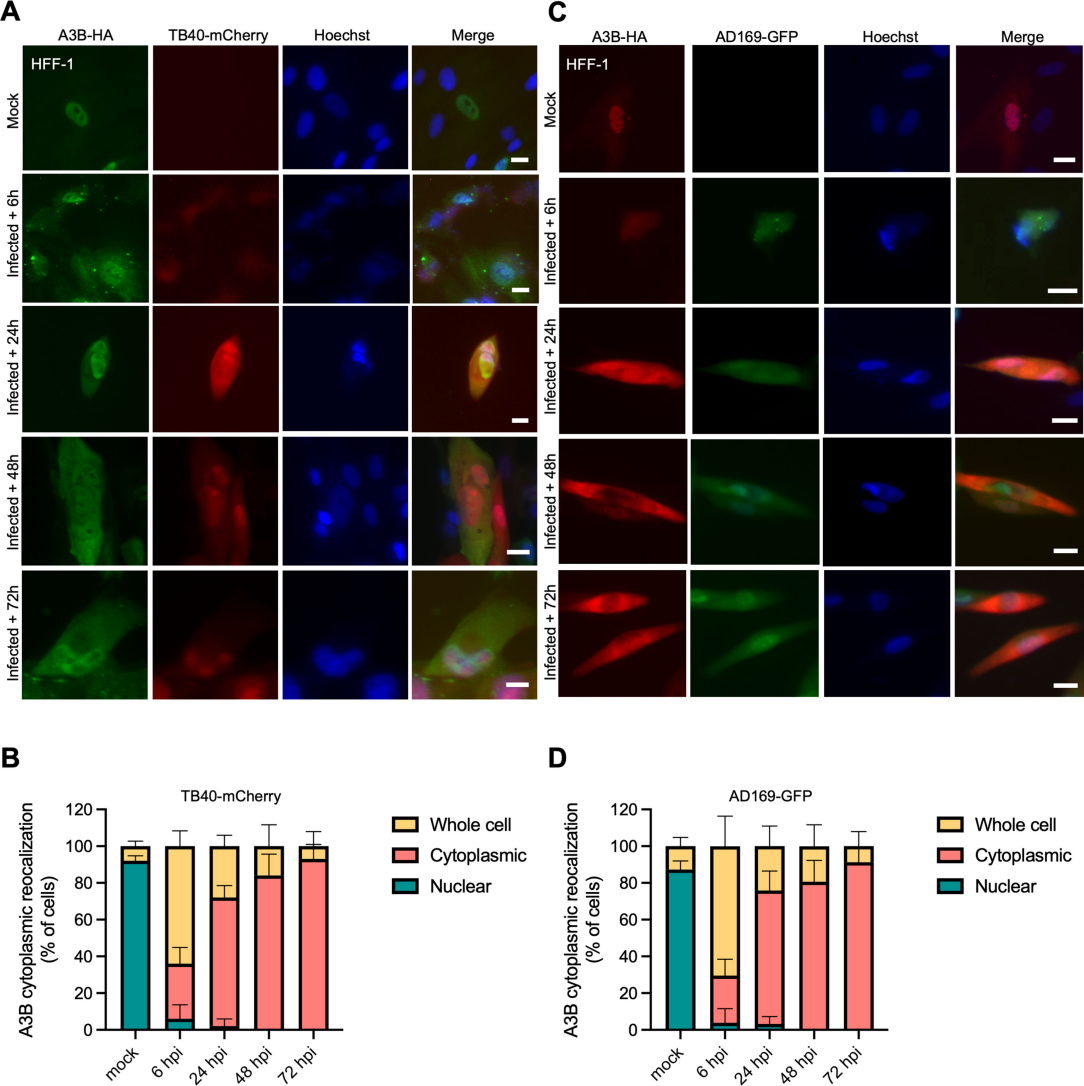
A3B relocalization occurs early during HCMV infection. (**A and C**) Representative IF microscopy images of HFF-1 cells stably expressing A3B-HA incubated with medium alone (mock) or infected with the indicated HCMV strains for the indicated timepoints (10 µm scale). (**B and D**) Quantification of A3B-HA subcellular localization phenotypes shown in panels A and C. Each histogram bar reports the percentage of cells with whole cell, cytoplasmic, and nuclear A3B-HA (*n* > 100 cells per condition; mean ± SD with *P* values from unpaired Student’s *t*-tests).

During a productive HCMV infection, viral genes are expressed chronologically in three main groups (28). IE genes are expressed at between 2 and 6 hpi, early (E) genes between 4 and 12 hpi, and late (L) genes following the onset of viral DNA replication (~24 hpi). To investigate the kinetics of A3B relocalization during HCMV infection, HFF-1 cells stably expressing A3B-HA were infected with TB40-mCherry or AD169-GFP and IF microscopy was performed at multiple timepoints after infection (6, 24, 48, and 72 hpi; Fig. 5A through D, respectively). This experiment shows that relocalization begins to occur rapidly with most infected cells exhibiting partial or full A3B-HA relocalization at the earliest timepoint 6 hpi. Moreover, the percentage of cells exhibiting cytoplasmic A3B-HA increases over time and is complete by 72 hpi. These kinetics suggest that an HCMV IE or E protein may be responsible for A3B relocalization during infection.

**Fig 6:**
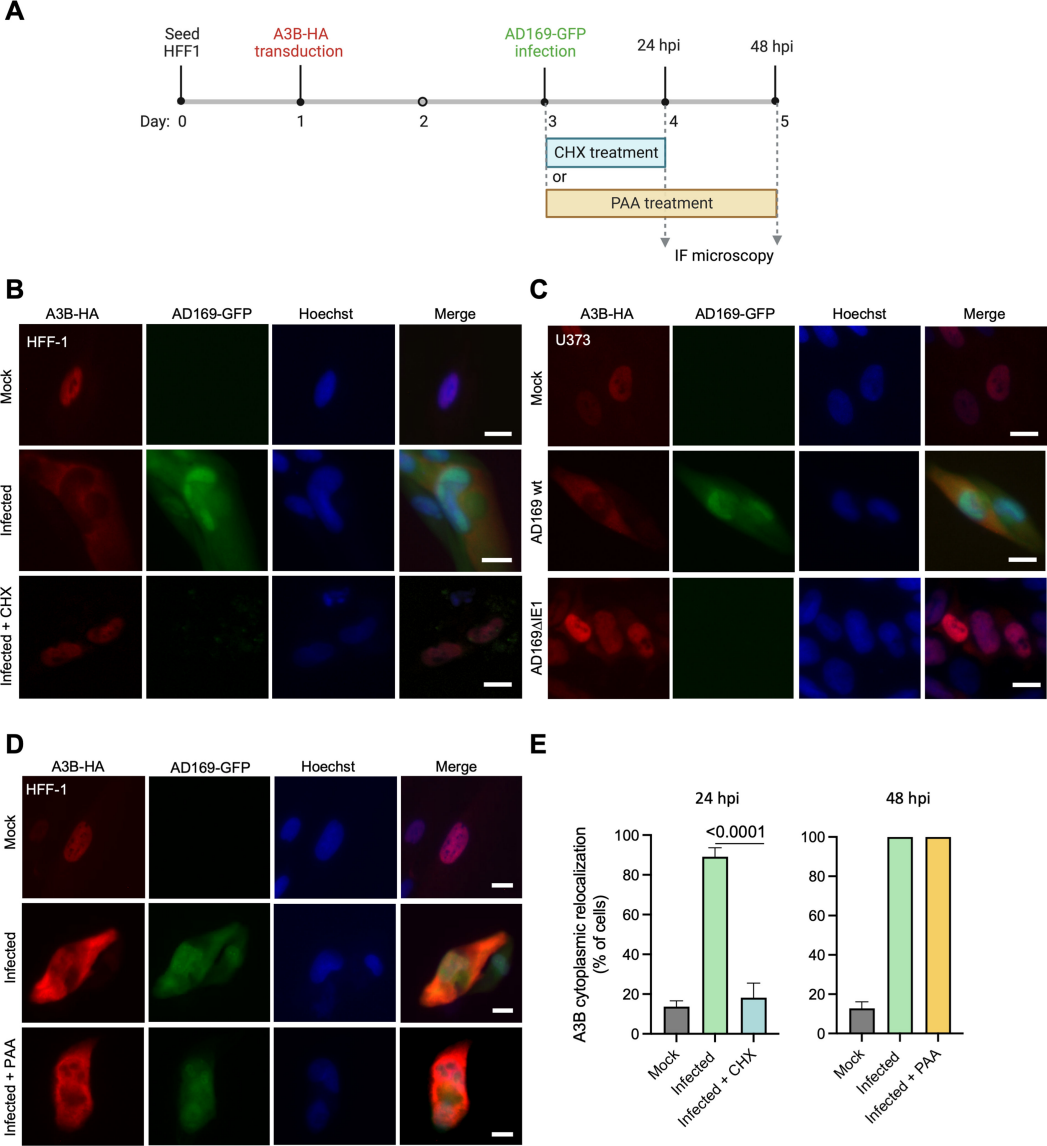
A3B relocalization requires *de novo* translation of HCMV proteins but not viral DNA synthesis. (**A**) Schematic representation of experimental workflows of CHX and PAA treatment of infected cells. Image created with BioRender. (**B**) Representative IF microscopy images of HFF-1 cells stably expressing A3B-HA incubated with medium alone (mock) or infected with AD169-GFP and treated with DMSO or CHX for 24 h (10 µm scale). (**C**) Representative IF microscopy images of U373 cells stably expressing A3B-HA incubated with medium alone (mock) or infected with AD169-GFP or AD169-GFP ΔIE1 for 72 h (10 µm scale). (**D**) Representative IF microscopy images of HFF-1 cells stably expressing A3B-HA incubated with medium alone (mock) or infected with AD169-GFP and treated with DMSO or PAA for 48 h (10 µm scale). (**E**) Quantification of A3B-HA subcellular localization phenotypes shown in panels B and D. Each histogram bar reports the percentage of cells with cytoplasmic A3B-HA (*n* > 80 cells per condition; mean ± SD with *P* values from unpaired Student’s *t*-tests).

To further investigate whether *de novo* viral protein expression is required for A3B relocalization, HFF-1 cells stably expressing A3B-HA were infected with AD169-GFP, treated for 24 h with the translation inhibitor cycloheximide (CHX) or dimethylsulfoxide (DMSO) as a control, and then subjected to IF microscopy (workflow in Fig. 6A). CHX treatment strongly prevents A3B-HA from relocalizing to the cytoplasm, whereas DMSO treatment does not (Fig. 6B and E). Similarly, cells infected with a recombinant AD169 lacking expression of the IE1 protein (AD169ΔIE1), which is required for expression of IE viral gene products (29), show no signs of A3B relocalization (Fig. 6C). In contrast, treating infected cells with phosphonoacetic acid (PAA), which blocks viral DNA synthesis and therefore also late protein expression (Fig. S4), fails to block A3B relocalization (Fig. 6D and E). Taken together with the rapid relocalization kinetics described above, these additional experiments implicate at least one HCMV IE/E protein in the A3B relocalization mechanism.

## DISCUSSION

The recent discovery that alpha- and gamma-herpesviruses have evolved strategies to escape from A3-mediated restriction suggested that the beta-herpesvirus HCMV might utilize a similar mechanism to counteract this potent innate immune defense system. Our results demonstrate that HCMV, similar to other herpesviruses, is able to alter the subcellular localization of the A3B enzyme, relocating it from the nucleus to the cytoplasm. However, this A3B relocalization mechanism is mechanistically distinct from other herpesvirus families, first, by occurring in an RNR-independent manner and, second, by targeting the regulatory NTD of A3B. In contrast, gamma- and alpha-herpesviruses utilize the large viral RNR subunit to bind to the catalytic CTD of A3B to mediate relocalization. Moreover, the rapid kinetics of A3B relocalization and pharmacologic (CHX) and genetic (IE1) requirements described above suggest the involvement of at least one IE/E viral gene product. These results combine to support a working model in which at least one HCMV IE/E protein binds to the regulatory NTD of A3B, promotes its relocalization to the cytoplasm, and thereby protects viral lytic DNA replication intermediates in the nucleus of the cell.

A3-mediated restriction of herpesviruses, including HCMV, has been reported by multiple groups (30 - 33). A3A is upregulated in HCMV-infected decidual tissues and associated with hypermutation of the viral genome (30). Another study reported that A3G is upregulated after HCMV infection of fibroblasts, even if the upregulation does not appear to modulate HCMV replication (32). These studies are certainly interesting, and our work here has not formally excluded these A3s in HCMV restriction. However, given that neither of these A3s appears to be counteracted by HCMV (i.e., degraded or relocalized), in contrast to A3B described here, they are not likely to pose a significant threat to viral genomic integrity *in vivo*. In contrast, A3B is relocalized away from sites of viral replication by HCMV, which suggests that it may be a *bona fide* threat to the virus during lytic replication. This possibility is supported by the preferred sites of A3B-mediated deamination (5’TC) being depleted from HCMV genomes, consistent with a longer-term evolutionary conflict between this enzyme and HCMV (33, 34). However, this likelihood is difficult to quantify experimentally until the factor involved in A3B neutralization is identified, mutated, and shown to be essential for virus replication in the presence (but not absence) of A3B.

A strength of the studies here is demonstrating A3B relocalization with multiple HCMV isolates and multiple different human cell types. Relocalization is also shown for endogenous A3B as well as epitope tagged constructs. The overall A3B cytoplasmic localization phenotype is similar to that of gamma- and alpha-herpesviruses except that it is an ambiguously RNR independent. However, a limitation to the studies here is an inability to determine whether the molecular mechanism involves active nuclear export of pre-existing A3B and/or a block to nuclear import of newly translated protein. Another limitation is the exclusive use of fluorescent microscopy for phenotypic determination. However, the speed at which relocalization is first detectable (6 hpi), pharmacologic inhibition through protein synthesis inhibition (CHX treatment), and genetic inhibition by preventing IE gene expression (ΔIE1) combine to support a model in which expression of at least one HCMV IE/E gene product is required for A3B relocalization. An alternative possibility is the involvement of a late gene product due to residual late gene expression following PAA treatment (Fig. S4). A major open question is, therefore, the identity of the viral factor(s) and potentially cellular co-factor(s) involved in this process.

Our studies here add HCMV to the list of herpesviruses that modulate A3B subcellular localization, suggesting that this function may be part of a counteraction mechanism essential for viral infection. Our studies are also consistent with the likelihood that this host–pathogen conflict is conserved evolutionarily (8, 11) with ancient origins and ongoing functionality to present day. It is surprising, however, that the mechanisms differ so dramatically on the molecular level such that HCMV (and perhaps other beta-herpesviruses) has evolved a distinct RNR-independent mechanism. If A3B neutralization proves essential for HCMV replication and pathogenesis, it may be possible in the future to drug the neutralization mechanism and enable natural restriction of viral infections.

## MATERIALS AND METHODS

### Cell culture

Cells were cultured at 37°C in a 5% CO2 atmosphere in a Thermo Forma incubator (Thermo Fisher Scientific, Waltham, MA, USA). HFF-1 (ATCC, Manassas, VA, USA), U373 (ATCC), and 293T cells were cultured in Dulbecco's Modified Eagle Medium (DMEM; Cytiva, Marlborough, MA, USA) supplemented with 10% fetal bovine serum (Gibco, Billings, MT, USA) and 1% penicillin/streptomycin (Gibco). ARPE19 cells (ATCC) were cultured in DMEM:F12 media (Gibco) supplemented with 10% fetal bovine serum (Gibco) and 1% penicillin/streptomycin (Gibco). HeLa cells (ATCC) were cultured in RPMI 1640 (Corning) supplemented with 10% fetal bovine serum (Gibco) and 1% penicillin/streptomycin (Gibco). All cells were checked periodically for *Mycoplasma* and they always tested negative.

### Viruses and infections

Viruses used in this study were as follows: TB40-mCherry [construction described in reference (35)]; AD169-GFP [construction described in reference (36)]; AD169-GFP-ΔUL45 [construction described in reference (26)]; and AD169ΔIE1 [construction described in reference (29)]. HCMV strain Merlin (GenBank accession NC 006273.2) was purchased from the ATCC. The strain FIX and its mutant FIXΔUL45 were a gift by Dr. Elena Percivalle (Fondazione IRCCS Policlinico San Matteo, Pavia, Italy) (37). The TB40-BAC4 and TB40-BAC4-UL45Stop strains used in Fig. 3E were produced using a markerless two-step RED-GAM recombination protocol (38, 39). To obtain the BAC of the mutant TB40/E UL45stop, the following primers were employed: UL45Stop_Fw: 5′- ATCTACCTGATTTCTTTGTTCTTTTCCTCGTAAACTTATGTAGACTCCGGCTGACGCGGACGAAGGATGACGACGATAAGTAGGG -3′; UL45Stop_Rv: 5′- CCGAGGACACCCGCTGTTCCTCGTCCGCGTCAGCCGGAGTCTACATAAGTTTACGAGGAAAAGCAACCAATTAACCAATTCTGATTAG -3′. All generated recombinant BAC DNAs were controlled for integrity and correctness by sequencing the mutated region. HFF-1 cells were used for the reconstitution of recombinant viruses and virus stock production. Viruses were then propagated by standard procedures as described (40). Briefly, HFF-1 cells were infected with a multiplicity of infection (MOI) of 0.01. When robust cytopathic effect was observed (between 7 and 14 d) cells were harvested. Then, centrifugation was performed at 15,000 × *g* for 30 min. Cell pellets were resuspended in complete media plus 15% sucrose phosphate buffer and sonicated on ice 4× for 10 s with 15 s between pulse. Centrifugation was performed at 1,300 × *g* for 5 min. Supernatant was collected, aliquoted, and frozen at −80°C. The viral titers were calculated using the 50% tissue culture infection dose method upon infection of HFF-1 cells with serially diluted viral supernatants. In all experiments, HFF-1, U373, and ARPE19 were infected with HCMV at an MOI of 3 PFU/cell by diluting the virus into the medium, allowing adsorption for 2 h and replacing the viral dilution with fresh medium.

### IF microscopy

For IF imaging of HCMV-infected cells, 5 × 10^4^ cells/well were seeded in a 24-well plate. After 24 h, cells were transduced with lentiviruses encoding for human A3B-HA or A3B-E255A-HA (Fig. 1A through E; Fig. 2A through E; Fig. 3D). 48 h after transduction, cells were infected with TB40-mCherry or AD169-GFP for up to 72 h as indicated in figure legends. In Fig. 6B and D, DMSO, CHX (100 µg/mL), or PAA (100 µg/mL) was added to the virus dilution, and after 2 h, when virus was removed, cells were incubated with fresh media and compounds for 24 h (CHX) and 48 h (PAA). Cells were fixed in 4% formaldehyde for 15 min, permeabilized in 0.2% Triton X-100 in phosphate-buffered saline (PBS) for 10 min, washed three times for 5 min in PBS, and incubated in blocking buffer [2.8 mM KH_2_PO_4_, 7.2 mM K_2_HPO_4_, 5% goat serum (Gibco), 5% glycerol, 1% cold water fish gelatin (Sigma, St Louis, MO, USA), 0.04% sodium azide (pH 7.2)] for 1 h. Cells were then incubated with primary rabbit anti-HA (1:2,000) (cat #3724, Cell Signaling, Danvers, MA, USA) or purified rabbit anti-A3B 5210-87-13 [1:300 (25); Fig. 2G], or mouse anti-HCMV-IE1 (1:2,000) (cat #MAB810R, EMD Millipore-Sigma, Burlington, MA, USA) (Fig. S2A) overnight at 4°C. Cells were washed three times for 5 min with PBS and then incubated with the secondary antibodies goat anti-rabbit IgG Alexa Fluor 488 (1:500) (cat #A11034, Invitrogen, Waltham, MA, USA), or goat anti-rabbit IgG Alexa Fluor 594 (1:500) (cat #A11037, Invitrogen), or goat anti-mouse IgG Alexa Fluor 488 (1:500) (cat #A11001, Invitrogen) for 2 h at room temperature in the dark. Cells were then counterstained with 1 µg/mL Hoechst 33342 for 20 min and rinsed twice for 5 min in PBS.

For IF imaging of transfected cells, 5 × 10^4^/well HeLa cells were transfected with plasmids expressing 200 ng pcDNA4-BORF2-FLAG or 200 ng pcDNA4-UL45-FLAG, and 100 ng pcDNA3.1-A3B-HA (Fig. 3C). 5 × 10^4^/well ARPE19 cells were transfected with plasmids expressing 100 ng pcDNA5TO-A3B-EGFP, pcDNA5TO-A3B-NTD-EGFP, pcDNA5TO-A3B-CTD-EGFP (Fig. 4A), and pcDNA4-BORF2-FLAG (Fig. 4C). Empty vector pcDNA3.1 or pcDNA3.1 encoding A3B-HA or other A3x-HA proteins is used in Fig. S1. After 48 h, IF was performed as described above. Cells were stained with primary antibodies mouse anti-FLAG (1:2,000) (cat #F1804, Sigma) and rabbit anti-HA (1:2,000) (cat #3724, Cell Signaling, Danvers, MA, USA) overnight at 4°C to detect FLAG-tagged RNRs and HA-tagged A3B, respectively. Goat anti-mouse IgG Alexa Fluor 488 (1:500) (cat #A11001, Invitrogen) and goat anti-rabbit IgG Alexa Fluor 594 (1:500) (cat #A11037, Invitrogen) were used as secondary antibodies.

Images in Fig. 1A (×60 magnification) and Fig. 3E (×20 magnification) were collected using an Eclipse Ti2 (Nikon). All other images were collected at ×20 magnification using an EVOS FL Cell Imaging System (Thermo Fisher Scientific). Scale bars are indicated in each panel and figure legend. Quantification was performed using Image J software, counting the percentage of cells with relocalized A3B or the ratio of nuclear/cytoplasmic A3B. Quantification was performed by counting cells from *n* = 3 independent experimental replicates. GraphPad Prism 9 was used to prepare graphs and statistical analyses (unpaired Student’s *t*-test).

### Co-IP experiments

293T (2.5 × 10^5^/well) cells were grown in 6-well plates and transfected with pcDNA3.1 plasmids encoding human A3A-HA, A3B-HA, and A3G-HA together or not with pcDNA4-BORF2-FLAG or pcDNA4-UL45-FLAG, and 6 µL TransIT-LT1 (Mirus, Madison, WI, USA) in 200 µL serum-free Opti-MEM (Thermo Fisher Scientific). After 48 h, whole cells were harvested in 300 µL of ice-cold lysis buffer [150 mM NaCl, 50 mM Tris-HCl, 10% glycerol, 1% IGEPAL (Sigma), and complete ethylenediaminetetraacetic acid (EDTA)-free protease inhibitor cocktail (Roche); pH 7.4]. Cells were vortexed, incubated on ice for 30 min, and then sonicated. Whole-cell lysates (30 µL) were aliquoted for input detection. Lysed cells were centrifuged at 13,000 rpm for 15 min to pellet debris, and the supernatant was resuspended with 25 µL anti-FLAG M2 magnetic beads (Sigma) for overnight incubation at 4°C with gentle rotation. Beads were washed three times in 700 µL of lysis buffer. Bound protein was eluted in 30 µL of elution buffer [0.15 mg/mL 3xFLAG peptide (Sigma) in 150 mM NaCl, 50 mM Tris-HCl, 10% glycerol, and 0.05% Tergitol; pH 7.4]. Input and eluted proteins were analyzed by immunoblots.

### Immunoblots

In Fig. 3A, membranes were stained with mouse anti-FLAG (1:5,000) (cat #3724, Sigma), mouse anti-tubulin (1:10,000) (cat # T5168, Sigma), and rabbit anti-HA (1:3,000) (cat #3724, Cell Signaling). After washing, membranes were incubated with an anti-rabbit IgG horseradish peroxidase-conjugated secondary antibody (1:10,000) (cat #211032171, Jackson ImmunoResearch, West Grove, PA, USA) and an anti-mouse IRDye 800CW (1:10,000) (cat #C70919-05, LI-COR, Lincoln, NE, USA).

In Fig. S4, membranes were stained with mouse anti-CMV pp65 antibody (cat #53489, Abcam, Cambridge, UK) (1:1,000) and mouse anti-tubulin (1:10,000) (cat # T5168, Sigma). Secondary antibody was a goat anti-mouse IRDye 680LT (1:10,000) (cat #926-68020, LI-COR).

### DNA deaminase activity assays

293T (5 × 10^5^/well) cells were seeded into 6-well plates and, after 24 h, transfected with 200 ng pcDNA4-BORF2-FLAG or 200 ng pcDNA4-UL45-FLAG, and 100 ng pcDNA3.1-A3B-HA. After 48 h, cells were harvested, resuspended in 100 µL of reaction buffer (25 mM HEPES, 15 mM EDTA, 10% glycerol, 1 tablet of Sigma-Aldrich cOmplete Protease Inhibitor Cocktail), and sonicated at the lowest setting. Whole-cell lysates were then centrifuged at 10,000 × *g* for 20 min. The clarified supernatant was incubated with 4 pmol of oligonucleotide (5′- ATTATTATTATTCAAATGGATTTATTTATTTATTTATTTATTT-fluorescein), 0.025 U uracil DNA glycosylase (UDG), 1x UDG buffer (NEB), and 1.75 U RNase A at 37°C for 2 h. Deamination mixtures were treated with 100 mM NaOH at 95°C for 10 min. Samples were then separated on 15% Tris-borate-EDTA-urea gel. Fluorescence was measured using a Typhoon FLA-7000 image reader (Fig. 3B).

## SUPPLEMENTAL MATERIAL

Supplemental figures jvi.00781-23-s0001.pdf Figures S1 to S4

ASM does not own the copyrights to Supplemental Material that may be linked to, or accessed through, an article. The authors have granted ASM a non-exclusive, world-wide license to publish the Supplemental Material files. Please contact the corresponding author directly for reuse.
