# Supplementary material for: Human cytomegalovirus mediates APOBEC3B relocalization early during infection through a ribonucleotide reductase-independent mechanism
Source: J Virol. 2023 Aug 11;97(8):e00781-23. doi: 10.1128/jvi.00781-23 (PMC10506462; doi:10.1128/jvi.00781-23)
Supplement: Supplemental figures — Figures S1 to S4 [file jvi.00781-23-s0001.pdf]

Supplementary Figs. S1-S4 for:

Human cytomegalovirus mediates APOBEC3B relocalization early during infection through a ribonucleotide reductase-independent mechanism

Elisa Fanunza[1.2], Adam Z. Cheng[3], Ashley A. Auerbach[1], Bojana Stefanovska[1,4], Sofia N. Moraes[3], James R. Lokensgard[5], Matteo Biolatti[6], Valentina Dell'Oste[6], Craig J. Bierle[7], Wade A. Bresnahan[8], Reuben S. Harris[1,4*]

[1] Department of Biochemistry and Structural Biology, University of Texas Health San Antonio, San Antonio, TX 78229, USA

[2] Department of Life and Environmental Sciences, University of Cagliari, Cittadella Universitaria di Monserrato, Monserrato (Cagliari), SS554, 09042, Italy

[3] Department of Biochemistry, Molecular Biology and Biophysics, University of Minnesota, Minneapolis, MN 55455, USA

[4] Howard Hughes Medical Institute, University of Texas Health San Antonio, San Antonio, TX 78229, USA

[5] Department of Medicine, University of Minnesota, Minneapolis, MN, 55455, USA

[6] Department of Public Health and Pediatric Sciences, University of Turin, Turin, 10126, Italy

[7] Department of Pediatrics, Division of Pediatric Infectious Diseases and Immunology, University of Minnesota, Minneapolis, MN 55455, USA

[8] Department of Microbiology and Immunology, University of Minnesota, MN 55455, USA

* Correspondence: rsh@uthscsa.edu

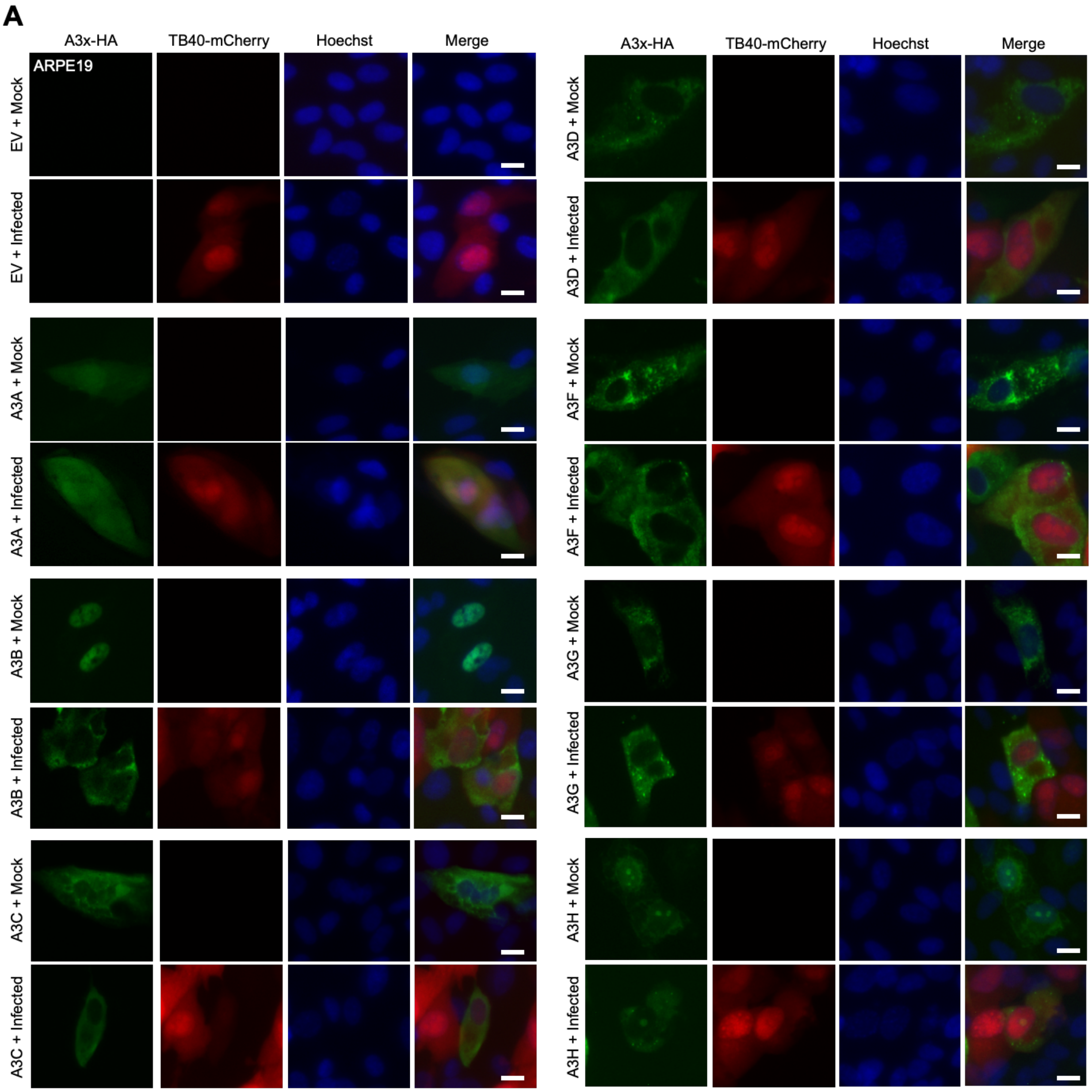

Fig. S1. Human A3 subcellular localization phenotypes following HCMV infection. Representative IF microscopy images of ARPE19 cells transfected with the indicated A3x-HA construct (green), infected with TB40-mCherry (red), and imaged 72 hpi (10 μm scale). All constructs encode wildtype human A3 proteins except A3A-E72A, which has a catalytic mutation to prevent toxicity.

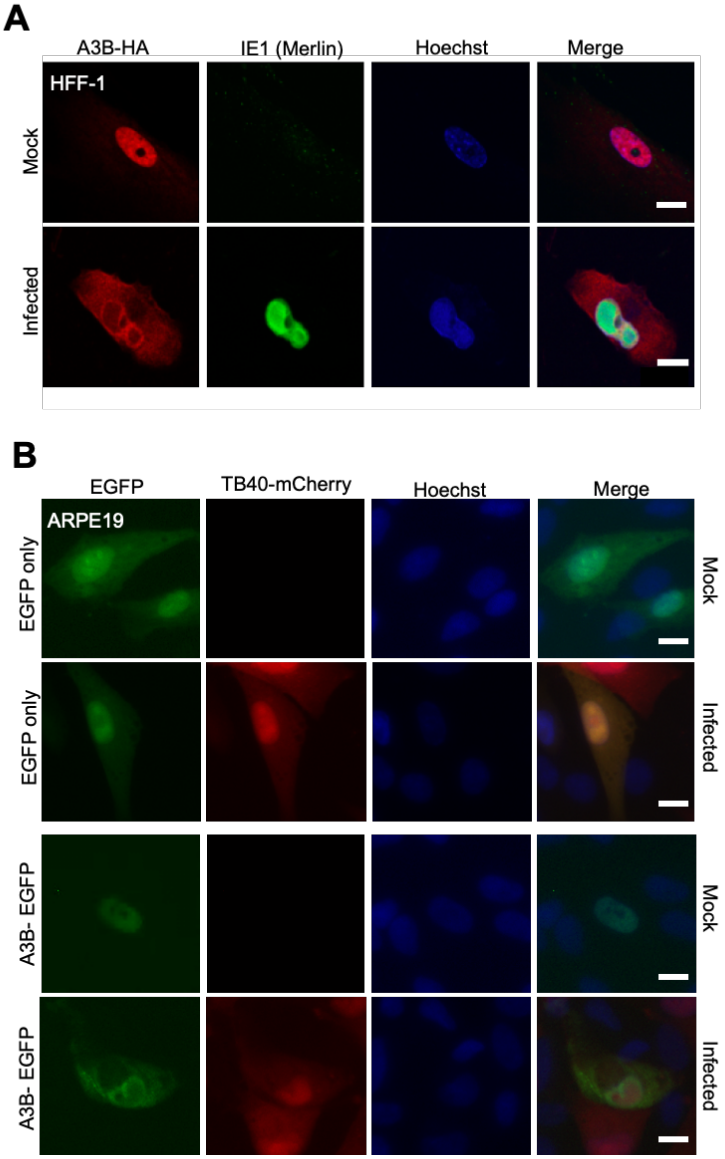

Fig. S2. A3B is relocalized by HCMV Merlin and TB40-mCherry.

(**A**) Representative IF microscopy images of HFF-1 cells expressing A3B-HA (red), mock or infected with Merlin strain (green) for 72 hrs (10 μm scale).

(**B**) Representative IF images of ARPE19 cells transfected with EGFP only or A3B-EGFP (green), mock or infected with TB40-mCherry (red) for 72 hrs (10 μm scale).

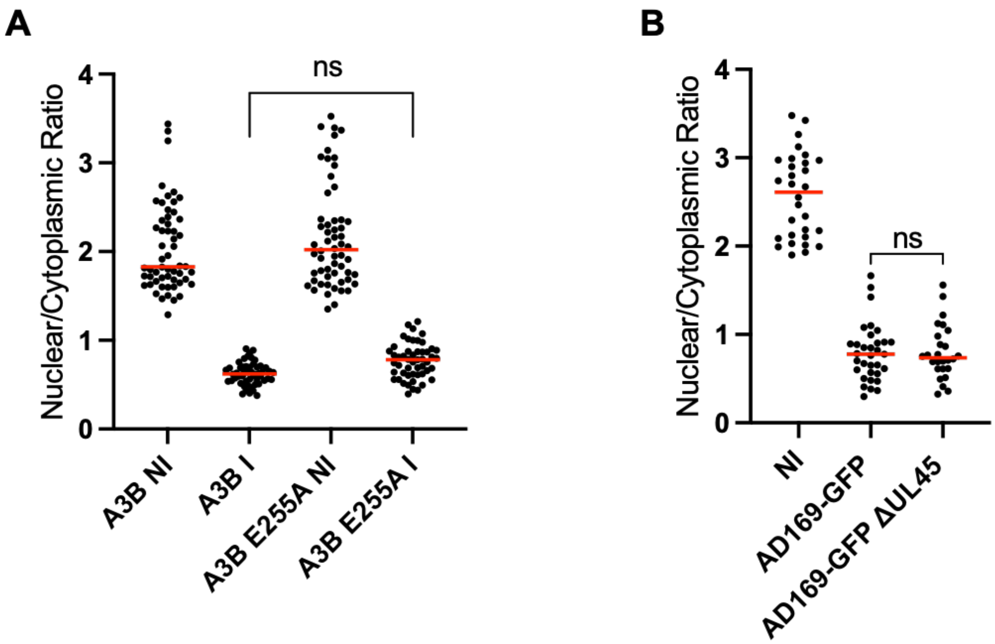

Fig. S3. Quantification of A3B and A3B-E255A relocalization phenotypes.

(**A**) HFF-1 stably expressing wt A3B or A3B-E255A were not infected (NI) or infected (I) with the AD169-GFP strain for 72 hrs. Quantification of Nuclear/Cytoplasmic Ratio of signal intensity for A3B or A3B-E255A with results showing that infection causes similarly strong relocalization phenotypes for both proteins (n>50 cells per condition; red lines are average values; ns, not significant, p = 0.34 by unpaired student's t-test).

(**B**) U373 stably expressing A3B or A3B-E255A were not infected (NI) or infected (I) with AD169 or AD169ΔUL45 for 72 hrs. Quantification of Nuclear/Cytoplasmic A3B Intensity Ratio, with both viruses triggering similar relocalization phenotypes (n>25 cells per condition; red lines are average values; ns, not significant, p = 0.94 by unpaired student's t-test).

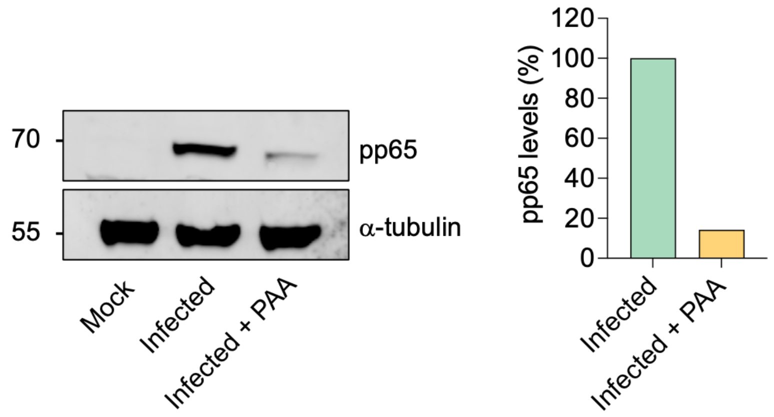

Fig. S4. PAA treatment blocks late protein accumulation.

Infected cells were treated or not with PAA for 48 hrs and then immunoblotting was used to evaluate expression levels of the late HCMV gene product pp65 (left, with band quantification to the right).
